# Left ventricular markers of global dyssynchrony predict limited exercise capacity in heart failure, but not in patients with preserved ejection fraction

**DOI:** 10.1186/1476-7120-10-36

**Published:** 2012-09-11

**Authors:** Gani Bajraktari, Arlind Batalli, Afrim Poniku, Artan Ahmeti, Rozafa Olloni, Violeta Hyseni, Zana Vela, Besim Morina, Rina Tafarshiku, Driton Vela, Premtim Rashiti, Edmond Haliti, Michael Y Henein

**Affiliations:** 1 Service of Cardiology, Clinic of Internal Medicine, University Clinical Centre of Kosova, Rrethi i Spitalit, p.n., Prishtina, Kosova; 2 Heart Centre and Department of Public Health and Clinical Medicine, Umeå University, Umeå, Sweden

**Keywords:** Six-minute walk test, Doppler echocardiography, LV function and dyssynchrony

## Abstract

**Background:**

The aim of this study was to prospectively examine echocardiographic parameters that correlate and predict functional capacity assessed by 6 min walk test (6-MWT) in patients with heart failure (HF), irrespective of ejection fraction (EF).

**Methods:**

In 147 HF patients (mean age 61 ± 11 years, 50.3% male), a 6-MWT and an echo-Doppler study were performed in the same day. Global LV dyssynchrony was indirectly assessed by total isovolumic time - t-IVT [in s/min; calculated as: 60 – (total ejection time + total filling time)], and Tei index (t-IVT/ejection time). Patients were divided into two groups based on the 6-MWT distance (Group I: ≤300 m and Group II: >300 m), and also in two groups according to EF (Group A: LVEF ≥ 45% and Group B: LVEF < 45%).

**Results:**

In the cohort of patients as a whole, the 6-MWT correlated with t-IVT (r = −0.49, p < 0.001) and Tei index (r = −0.43, p < 0.001) but not with any of the other clinical or echocardiographic parameters. Group I had lower hemoglobin level (p = 0.02), lower EF (p = 0.003), larger left atrium (p = 0.02), thicker interventricular septum (p = 0.02), lower A wave (p = 0.01) and lateral wall late diastolic myocardial velocity a’ (p = 0.047), longer isovolumic relaxation time (r = 0.003) and longer t-IVT (p = 0.03), compared with Group II. In the patients cohort as a whole, only t-IVT ratio [1.257 (1.071-1.476), p = 0.005], LV EF [0.947 (0.903-0.993), p = 0.02], and E/A ratio [0.553 (0.315-0.972), p = 0.04] independently predicted poor 6-MWT performance (<300 m) in multivariate analysis. None of the echocardiographic measurements predicted exercise tolerance in HFpEF.

**Conclusion:**

In patients with HF, the limited exercise capacity, assessed by 6-MWT, is related mostly to severity of global LV dyssynchrony, more than EF or raised filling pressures. The lack of exercise predictors in HFpEF reflects its multifactorial pathophysiology.

## Background

Heart failure (HF) has become a major public health problem [1], and its incidence is increasing [2], particularly that caused by systolic left ventricular (LV) dysfunction, which is known to have poor prognosis [3,4]. In patients with systolic HF, systolic [5] and diastolic [6,7] LV velocity parameters, as well as right ventricular [8] function have been shown to correlate with functional capacity. However, detailed assessment of cardiac function timing [9] has shown that similar clinical limitation could be seen in patients with isolated diastolic myocardial abnormalities in the presence of preserved LV ejection fraction (EF) (HFpEF) [10]. Irrespective of EF, the morbidity and mortality of patients with HF remain high, despite advances in pharmacological and non-pharmacological treatments [11,12], suggesting a need for identifying other functional parameters that guide towards optimum management and better clinical outcome.

Ventricular dyssynchrony has been shown to contribute to clinical deterioration of HF and resynchronization therapy has proved the opposite [13-15]. LV segmental and global dyssynchrony can easily be assessed by Doppler echocardiography [16-18], with its parameters not only predicting prognosis but also the extent of exercise tolerance. Six-minute walk test (6-MWT) is very popular for objective assessment of exercise capacity in HF [19], particularly in secondary and tertiary referral centers [20,21]. We have previously shown that echocardiographic markers of raised left atrial pressure and those of ventricular dyssynchrony predict limited exercise capacity in patients with poor LV EF [22]. However, the predictive role of echocardiography in assessing limited exercise capacity in patients with HFpEF has not been thoroughly investigated. The aim of this study was to prospectively examine predictors of functional capacity, assessed by 6-MWT in a consecutive group of patients irrespective of EF. This objective is based on the rationale that HFpEF patients have multi-factorial causes for limited exercise capacity therefore difficult to identify breathlessness related mechanisms using Doppler echocardiography.

## Methods

### Study population

We studied 147 patients (mean age 61 ± 11 years, 50.3% male) with clinical diagnosis of congestive heart failure, and New York Heart Association (NYHA) functional class I-III, secondary to ischemic heart disease or non-ischemic etiology. Patients were referred to the Service of Cardiology, Internal Medicine Clinic, University Clinical Centre of Kosovo, between December 2005 and April 2011. At the time of the study all patients were on optimum cardiac medications, optimized at least 2 weeks prior to enrollment, based on patient’s symptoms and renal function: 81% were receiving ACE inhibitors or ARB, 70% beta-blockers, 11% digoxin, 46% spironolactone, 64% diuretics. Patients with reduced LV EF had ischemic etiology in 42%, hypertensive in 25%, and unknown etiology in 33%. Patients with preserved LV EF had ischemic aetiology in 44% and hypertensive in 56%. All patients were in sinus rhythm. Fifty seven patients had LV EF <45% (HFpEF), and the remaining 90 patients had LV EF ≥45% (Table 1). Patients with clinical evidence for cardiac decompensation, limited physical activity due to factors other than cardiac symptoms (e.g. arthritis), more than mild mitral regurgitation, more than mild renal failure, chronic obstructive pulmonary disease (COPD) or those with recent acute coronary syndrome, stroke or anemia were excluded. Patients gave a written informed consent to participate in the study, which was approved by the local Ethics Committee.

**Table 1:** Comparison of echocardiographic data between patient’s groups

***Variable***	***Limited performance***	***Good performance***	***P***
	***(n = 90)***	***(n =57)***	
***Systolic LV function***			
Ejection fraction (%)	34 ± 12	41 ± 15	0.003
Interventricular septum (cm)	1.06 ± 0.1	1.11 ± 0.2	0.07
Left atrium (cm)	4.8 ± 0.7	4.5 ± 0.7	0.02
LV EDD (cm)	6.4 ± 1.3	6.1 ± 1.1	0.15
LV ESD (cm)	5.3 ± 1.0	4.9 ± 1.1	0.08
Septal long axis amplitude (cm)	0.8 ± 0.3	0.9 ± 0.3	0.21
Septal s’ wave (cm/s)	6.3 ± 1.4	7 ± 2.6	0.24
Lateral long axis amplitude (cm)	1.05 ± 0.3	1.1 ± 0.3	0.24
Lateral s’ wave (cm/s)	6.1 ± 2.4	6.5 ± 2.5	0.51
LV posterior wall (cm)	1.0 ± 0.1	1.0 ± 0.4	0.86
Aortic root (cm)	3.4 ± 0.3	3.4 ± 0.4	0.94
***Diastolic LV function***			
A wave velocity (cm/s)	58 ± 21	72 ± 29	0.009
Lateral a’ wave (cm/s)	7 ± 2.5	8.3 ± 3.5	0.047
E wave deceleration time (ms)	146 ± 57	168 ± 65	0.08
Lateral e’ wave (cm/s)	6 ± 2.3	6.4 ± 2	0.28
E/A ratio	1.2 ± 0.8	1.6 ± 1.1	0.045
Septal a’ wave (cm/s)	8.2 ± 2.5	8.7 ± 5	0.64
Septal e’ wave (cm/s)	6 ± 2.2	6 ± 2.8	0.71
E wave velocity (cm/s)	69 ± 24	70 ± 32	0.75
Left atrial area	27 ± 8.2	28 ± 8.6	0.88
***Global LV function***			
T-IVT	14.1 ± 3.8	11.8 ± 4.4	0.03
Tei index	0.74 ± 0.5	0.6 ± 0.3	0.07
IVRT	121 ± 35	99 ± 24	0.003
E/e’ ratio	12.6 ± 6.6	11.6 ± 5.6	0.41
***RV function***			
A wave velocity (cm/s)	51 ± 15	54 ± 22	0.44
E wave deceleration time (ms)	154 ± 68	157 ± 71	0.84
Right long axis amplitude (cm)	2 ± 0.61	2 ± 0.6	0.93
PSAP (mmHg)	44.3 ± 23	44.4 ± 17	0.99
Right e’ wave (cm/s)	9.7 ± 4.3	9 ± 3.8	0.57
Right a’ wave (cm/s)	15 ± 5.6	13 ± 4.7	0.24
Right s’ wave (cm/s)	10.6 ± 2.9	11.2 ± 3.2	0.52
EDD (cm)	3.2 ± 0.9	2.8 ± 0.6	0.06

*LV* left ventricle, *RV* right ventricle, *A* atrial diastolic velocity, *E* early diastolic filling velocity, *EDD* end-diastolic dimension, *ESD* end-systolic dimension, *T-IVT* total isovolumic time, *IVRT* isovolumic relaxation time, *s’* systolic myocardial velocity, *e’* early diastolic myocardial velocity, *a’* late diastolic myocardial velocity.

### Data collection

Detailed history and clinical assessment were obtained in all patients, in whom routine biochemical tests were also performed including hemoglobin, lipid profile, blood glucose level, and kidney function tests. Estimated body mass index (BMI) was calculated from weight and height measurements. Waist, hip measurements were also made and waist/hip ratio calculated.

### Echocardiographic examination

A single operator performed all echocardiographic examinations using a Philips Intelligent E-33 system with a multi-frequency transducer, and harmonic imaging as appropriate. Images were obtained with the patient in the left lateral decubitus position and during quiet expiration. End-systole and end-diastole LV dimensions were made from the left parasternal long axis view with the M-mode cursor positioned by the tips of the mitral valve leaflets. LV volumes and EF were calculated from the apical 2 and 4 chamber views using the modified Simpson’s method. Ventricular long axis motion was studied by placing the M-mode cursor at the lateral and septal angles of the mitral ring and the lateral angle of the tricuspid ring. Total amplitude of long axis motion was measured as previously described [23] from peak inward to peak outward points. LV and right ventricular (RV) long axis myocardial velocities were also studied using Doppler myocardial imaging technique. From the apical 4-chamber view, longitudinal velocities were recorded with the sample volume placed at the basal part of LV lateral and septal segments as well as RV free wall. Systolic (s’), as well as early and late (e’ and a’) diastolic myocardial velocities were measured with the gain optimally adjusted. Mean value of the lateral and septal LV velocities were calculated. Left atrial diameter was measured from aortic root recordings with the M-mode cursor positioned at the level of the aortic valve leaflets.

Diastolic function of the LV and RV was assessed from filling velocities using spectral pulsed wave Doppler with the sample volume positioned at the tips of the mitral and tricuspid valve leaflets, respectively, during a brief apnea. Peak LV and RV early (E wave), and late (A wave) diastolic velocities were measured and E/A ratios were calculated. The E/e’ ratio was calculated from the transmitral E wave and the mean lateral and septal segments e’ waves. The isovolumic relaxation time was also measured from aortic valve closure to mitral valve opening, on the pulsed wave Doppler recording. LV filling pattern was considered ‘restrictive’ when E/A ratio was >2.0, E wave deceleration time < 140 ms and the left atrium dilated of more than 40 mm in transverse diameter [24].

### Measurements of LV dyssynchrony

Indirect assessment of LV dyssynchrony was obtained by measuring total isovolumic time (t-IVT), Tei Index and LV-RV pre-ejection time delay. Total LV filling time was measured from the onset of the E wave to the end of the A wave and ejection time from the onset to the end of the aortic Doppler flow velocity. Total isovolumic time (t-IVT) was calculated as 60 - (total ejection time + total filling time) and was expressed in s/min [25]. Tei index was calculated as the ratio between t-IVT and ejection time [26]. LV and RV pre-ejection times were measured as the time interval between the onset of the q wave and the onset of the aortic and pulmonary forward flow velocities, respectively and the time delay between them was calculated [26].

Mitral regurgitation severity was assessed by color and continuous wave Doppler and was graded as mild, moderate, or severe according to the relative jet area to that of the left atrium as well as the flow velocity profile, in line with the recommendations of the American Society of Echocardiography [27]. Likewise, tricuspid regurgitation was assessed by color Doppler and continuous-wave Doppler. Retrograde trans-tricuspid pressure drop > 35 mmHg was taken as an evidence for pulmonary hypertension [28]. All M-mode and Doppler recordings were made at a fast speed of 100 mm/s with a superimposed ECG (lead II).

### Six minute walk test

Within 24 h of the echocardiographic examination a 6-MWT was performed on a level hallway surface for all patients and was administered by a specialized nurse blinded to the results of the echocardiogram. According to the method of Gyatt et al. [29] patients were informed of the purpose and protocol of the 6 MWT which was conducted in a standardized fashion while patients on their regular medications [30,31]. A 15 m flat, obstacle-free corridor was used and patients were instructed to walk as far as they can, turning 180° after they have reached the end of the corridor, during the allocated time of 6 min. Patients walked unaccompanied so as not to influence walking speed. At the end of the 6 min the supervising nurse measured the total distance walked by the patient. Pulse and blood pressure were measured before and at the end of the walking test.

#### Statistical analysis

Data are presented as mean ± SD or proportions (% of patients). Continuous data was compared with two-tailed unpaired Student’s *t* test and discrete data with Chi-square test. Correlations were tested with Pearson coefficients. Predictors of 6 MWT distance were identified with univariate analysis and multivariate logistic regression was performed using the step-wise method, a significant difference was defined as P < 0.05 (2-tailed). Patients were divided according to their ability to walk >300 m into good and limited exercise performance groups [32], and were compared using unpaired Student *t*-test. Also, patients with maintained HFpEF were compared with those with reduced EF (<45%) using unpaired *t*-test.

## Results

Patients mean age was 61 ± 11 years, and 50.3% were females (Table 2). The etiology of heart failure was ischemic in 68 patients (46%), idiopathic in 44 (30%) and hypertensive in 35 (24%) patients. The studied patients as a whole exercised for a mean of 265 ± 111 m and had to stop because of breathlessness and/or tiredness. Patients with HF and reduced EF exercised for 241 ± 107 m compared to 275 ± 112 m in those with HFpEF, (p = 0.09).

**Table 2:** Baseline patient’s data in study patients

	
Sex (female, in%)	49.7
Age (years)	61 ± 11
Smoking (%)	31
Diabetes (%)	33
LBBB (%)	25
Body-mass index	28 ± 5
Waist/hips ratio	0.95 ± 0.1
NYHA class	2.3 ± 0.6
Fasting glucose (mmol/L)	7 ± 3.3
Total cholesterol (mmol/L)	4.3 ± 1.3
Triglycerides (mmol/L)	1.7 ± 1
Urea (mmol/L)	9.9 ± 4.5
Creatinine (μmol/L)	109 ± 41
Hemoglobine (g/L)	128 ± 23
Heart rate (beats/min)	78 ± 13

### Clinical and echocardiographic correlates of 6 MWT distance

**Figure 1:**
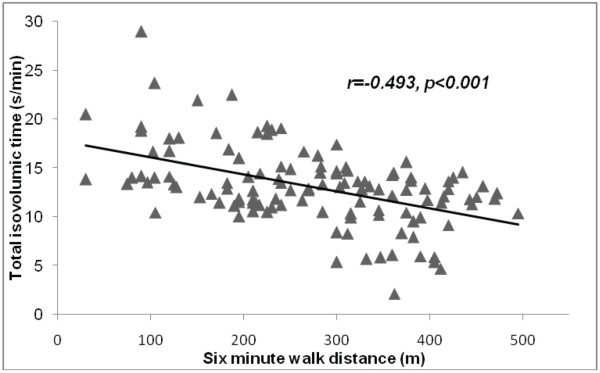
Correlation between total isovolumic time and 6 min walk distance in patients with heart failure.

**Figure 2:**
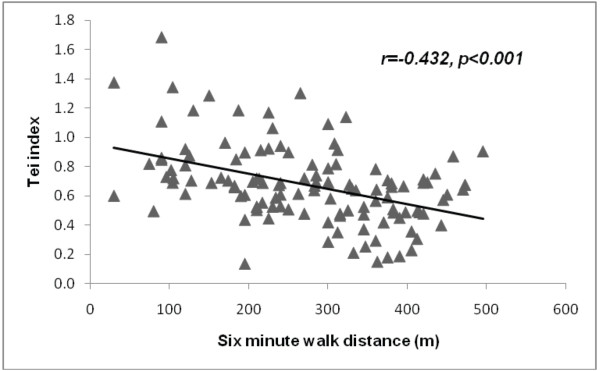
Correlation between Tei index and 6 min walk distance in patients with heart failure.

Out of the list of Doppler echocardiographic measurements, only markers of global dyssynchrony, t-IVT (r = −0.49, p < 0.001) and Tei index (r = −0.43, p < 0.001) correlated with the 6-MWT distance using Pearson’s correlation model (Figure 1 &2).

### Patients with limited vs. good 6 MWT performance

Hemoglobin (p = 0.03) and smoking (p = 0.04) were the only clinical finding that was different between the two groups of patients with good and limited exercise performance (Table 3). Patients with limited 6-MWT performance had lower EF (p = 0.003), larger left atrium (p = 0.02), lower A wave (p = 0.009) and lateral myocardial velocity a’ (p = 0.047), but longer isovolumic relaxation time (p = 0.003) and longer t-IVT (p = 0.03, Table 1).

**Table 3:** Comparison of clinical and biochemical data between patient’s groups

***Variable***	***Limited performance***	***Good performance***	***P value***
	***(n = 90)***	***(n =57)***	
Sex (female, in%)	53	43	0.26
Age (years)	61 ± 12	61 ± 10	0.81
Smoking (%)	25	44	0.04
Diabetes (%)	34	30	0.67
LBBB (%)	24	26	0.75
Preserved EF (%)	30	40	0.24
Body-mass index	28 ± 5	28 ± 4	0.91
Waist/hips ratio	0.95 ± 0.1	0.97 ± 0.1	0.28
NYHA class	2.3 ± 0.6	2.5 ± 0.7	0.09
Fasting glucose (mmol/L)	6.9 ± 3.3	7.1 ± 2.7	0.84
Total cholesterol (mmol/L)	4.2 ± 1.2	4.3 ± 1.3	0.16
Triglycerides (mmol/L)	1.8 ± 1.2	1.6 ± 0.4	0.25
Urea (mmol/L)	9.9 ± 4.3	9.5 ± 5.2	0.66
Creatinine (μmol/L)	111 ± 36	108 ± 55	0.74
Haemoglobine (g/L)	123 ± 28	133 ± 16	0.03
Heart rate (beats/min)	79 ± 13	77 ± 14	0.51

Data are mean ± standard deviation. NYHA = New York Heart Association.

### Predictors of limited 6 MWT distance

#### Univariate predictors of limited 6 MWT distance

From the biochemical and clinical findings, only low haemoglobin level (p = 0.047) predicted limited 6-MWT distance. Prolonged t-IVT (p < 0.001), high Tei index (p < 0.001), prolonged isovolumic relaxation time (p = 0.005), low LV EF (p = 0.007) and high E/A ratio (p = 0.03), were the echocardiographic predictors of limited distance (Table 4).

**Table 4:** Predictors of limited 6 min walk test

***Variable***	***Odds ratio (95***% ***CI)***	***P value (<)***
***Clinical univariate predictors***		
Hemoglobin	1.020 (1.000-1.041)	0.047
Heart rate	1.027 (0.999-1.056)	0.06
NYHA class	1.535 (0.878-2.685)	0.13
Gender	1.502 (0.734-3.073)	0.27
Creatinine	1.003 (0.995-1.012)	0.47
Urea	1.030 (0.950-1.117)	0.47
Body-mass index	1.028 (0.947-1.117)	0.51
Diabetes mellitus	0.847 (0.394-1.820)	0.67
Age	1.004 (0.973-1.036)	0.81
**Echocardiographic univariate predictors**		
T-IVT	0.743 (0.646-0.855)	<0.001
Tei index	0.038 (0.007-0.219)	<0.001
IVRT	0.974 (0.957-0.992)	0.005
LV EF	1.049 (1.013-1.087)	0.007
E/A ratio	1.526 (1.035-2.251)	0.03
LV EDD	0.714 (0.483-1.055)	0.09
Right long axis amplitude	1.832 (0.885-3.791)	0.11
LV ESD	0.781 (0.562-1.086)	0.14
Left atrium	1.272 (0.763-2.120)	0.36
E wave deceleration time	0.997 (0.991-1.003)	0.39
E/e’ ratio	1.036 (0.943-1.140)	0.46
Lateral long axis amplitude	0.708 (0.197-2.554)	0.59
Septal long axis amplitude	0.794 (0.857-3.404)	0.76
**Multivariate predictors**		
T-IVT	1.257 (1.071-1.476)	0.005
LV Ejection Fraction	0.947 (0.903-0.993)	0.02
E/A ratio	0.553 (0.315-0.972)	0.04
Haemoglobin	0.974 (0.947-1.001)	0.06
Gender	0.535 (0.188-1.523)	0.24
Age	1.022 (0.975-1.072)	0.37

*LV* left ventricle, *A* atrial diastolic velocity, *E* early diastolic filling velocity, *EDD* end-diastolic dimension, *ESD* end-systolic dimension, *T-IVT* total isovolumic time, *IVRT* isovolumic relaxation time, *s’* systolic myocardial velocity, *e’* early diastolic myocardial velocity, *a’* late diastolic myocardial velocity.

#### Multivariate predictors of limited 6 MWT distance

**Figure 3:**
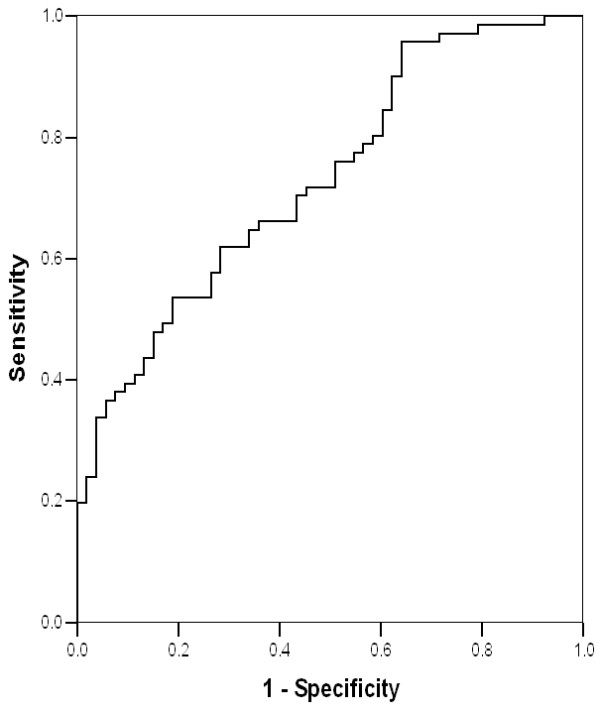
ROC-curve of t-IVT in predicting poor exercise performance on 6-min walk test in patients with heart failure.

In multivariate analysis [odds ratio 95% confidence interval], prolonged t-IVT [1.257 (1.071-1.476), p = 0.005], low LV EF [0.947 (0.903-0.993), p = 0.02], and high E/A ratio [0.553 (0.315-0.972), p = 0.04], independently predicted the limited 6-MWT distance (Table 4). A t-IVT of 12.5 s/min had a sensitivity of 70% and specificity of 65% (AUC on ROC analysis of 73%) for predicting limited performance (Figure 3).

#### *Patients with EF < 45*% *vs patients with HFpEF*

We further divided the whole cohort into patients with reduced EF (<45%) and those with HFpEF and compared their clinical and echocardiographic parameters, as well as exercise capacity and its predictors.

Heart rate was the only clinical variable slightly lower in patients with HFpEF compared to those with low EF (p = 0.04). LV EDD, LV ESD and left atrium were all smaller in HFpEF patients (p < 0.001, for all). Interventricular septum was thicker (p = 0.01) and septal long axis amplitude of motion (p = 0.049) were both higher in HFpEF patients. LV filling A wave was higher (p = 0.002), E wave deceleration time longer p = 0.03, and E/A ratio lower (p = 0.008) in HFpEF patients (Table 1). None of clinical or echocardiographic parameters independently predicted the limited exercise in patients with HFpEF.

## Discussion

### Findings

In this study we identified markers of global LV dyssynchrony, measured by t-IVT and Tei index but not pre-ejection time which correlated with the 6 MWT distance in a group of patients with clinically stable HF irrespective of EF. A long t-IVT, a low LV EF and high trans-mitral E/A ratio predicted limited exercise performance in multivariate analysis. Low hemoglobin, a univariate predictor, failed to independently predict limited exercise performance. In a small subgroup of patients with HFpEF none of the clinical parameters or functional echocardiographic measurements predicted limited 6MWT distance.

### Data interpretation

In the absence of musculoskeletal disorders, increased body weight or chronic obstructive pulmonary disease, limited exercise performance is commonly related to cardiac dysfunction, in the form of reduced systolic function (EF) or raised filling pressures [22,33]. These two common findings have similar consequences, in the form of raised pulmonary pressure, causing exertional breathlessness. Our studied patients show this disturbed pathophysiology, even partially, having shown reduced LV EF and global LV dyssynchrony as independent predictors of limited exercise performance. Global dyssynchrony in the form of prolonged t-IVT and high Tei index restrict effective phases of the cardiac cycle, required for LV filling and ejection. This results in compromised stroke volume and consequently cardiac output and exercise performance as previously shown [33]. In addition, limited left atrial emptying is bound to increase pulmonary venous pressure and hence breathlessness [34] and limited exercise capacity. In individual patients, a t-IVT cut off value of 12.5 s/m clearly discriminated good from limited exercise capacity, having identified over 50% of patients. It must be mentioned that normal exercise capacity is dictated by limited stroke volume or raised left atrial pressure, based on gender [35]. Theoretically therefore, correcting LV synchronous function in heart failure patients by cardiac resynchronisation therapy (CRT), in the absence of evidence for a need for revascularization, is bound to reverse such disturbed physiology and cardiac symptoms [36]. On the other hand when segregating the cohort into patients with reduced EF and those with HFpEF such predictive power of cardiac measurements for 6MWT distance was lost, suggesting the presence of other mechanisms for breathlessness, even in a minority of patients, which diluted the relationship we found above. It also confirms the lack of uniformity of such patients, currently combined under one diagnosis ‘HFpEF’ as has recently been shown not to respond to one and the same medical protocol [37]. This finding confirms our hypothesis stated in the introduction above.

### Limitations

We did not obtain Doppler echocardiographic measurements at peak exercise, when patients became symptomatic, since the objective of the study was to simply determine predictors of ordinary walking exercise limitation rather than an exercise echocardiogram that should have required a different protocol. We did not have invasive measurements of left atrial pressures but relied on Doppler measurements, which are known to be highly reproducible and to closely correlate with invasive pressure measurements [38]. We avoided using segmental measurements of dyssynchrony, based on the recent doubt of their accuracy [31]. We did not have BNP ant NT-pro-BNP data in our patients. However, as the aim of the study was the assessment of echocardiographic predictors of exercise capacity in heart failure patients, we believe that the lack of these data will not weaken the results.

### Clinical implications

Patients with HF, particularly those in NYHA class III and IV are known to have significantly limited exercise tolerance. Although EF is the most popular measure of LV function, and is considered the corner stone for recruiting patients for various treatment modalities, markers of global LV dyssynchrony should be considered as part of the conventional protocol of the follow-up of such patients. Despite exertional breathlessness in HF patients is multi-factorial, identifying specific patterns of cardiac dysfunction should guide towards optimum management of individual patients, e.g. vasodilators in patients with raised left atrial pressure and cardiac resynchronisation therapy in those with ventricular dyssynchrony, particularly when there is evidence for irreversible dyssynchrony. Those with HFpEF need individualistic approach in assessing their exercise capacity and management.

## Conclusions

Patients with heart failure who appear to be clinically stable may have significantly limited exercise ability. In these patients, markers of global ventricular dyssynchrony are as important, if not more, than markers of systolic LV dysfunction, in predicting limited exercise performance. Results of the small subgroup of patients with HFpEF cannot be generalised without being tested in a larger cohort.

## Competing interest

The authors declare that they have no competing interests.

## Authors contributions

GB, EH and MH designed the study; GB, AB, AP, AA, RO and ZV, made the protocol of study; GB, VH, BM, PR, DV, RT and EH, examined the patients; AB, AA, AP, EH, ZV, RO, PR, RT, DV and BM wrote the paper. GB and MH made critical review of the paper; All authors read and approved the final manuscript.

## References

[B1] Lloyd-Jones D Adams R Carnethon M De Simone G Ferguson TB Flegal K Ford E Furie K Go A Greenlund K Haase N Hailpern S Ho M Howard V Kissela B Kittner S Lackland D Lisabeth L Marelli A McDermott M Meigs J Mozaffarian D Nichol G O'Donnell C Roger V Rosamond W Sacco R Sorlie P Stafford R Steinberger J Thom T Wasserthiel-Smoller S Wong N Wylie-Rosett J Hong Y American heart association statistics committee and stroke statistics subcommittee. heart disease and stroke statistics-2009 update: a report from the American heart association statistics committee and stroke statistics subcommittee Circulation 2009 119 480 6 19171871 10.1161/CIRCULATIONAHA.108.191259

[B2] Weir RA McMurray JJ Epidemiology of heart failure and left ventricular dysfunction after acute myocardial infarction Curr Heart Fail Rep 2006 3 175 80 10.1007/s11897-006-0019-5 17129511

[B3] Davies M Hobbs F Davis R Kenkre J Roalfe AK Hare R Wosornu D Lancashire RJ Prevalence of left-ventricular systolic dysfunction and heart failure in the Echocardiographic Heart of England Screening study: a population based study Lancet 2001 358 439 45 10.1016/S0140-6736(01)05620-3 11513906

[B4] Ho KK Anderson KM Kannel WB Grossman W Levy D Survival after onset of congestive heart failure in Framingham Heart Study subjects Circulation 1993 88 107 15 10.1161/01.CIR.88.1.107 8319323

[B5] Ciampi Q Pratali L Porta MD Petruzziello B Manganiello V Villari B Picano E Sicari R Tissue Doppler systolic velocity change during dobutamine stress echocardiography predicts contractile reserve and exercise tolerance in patients with heart failure Eur Heart J Cardiovasc Imaging 2012 [Epub ahead of print] 10.1093/ehjci/jes096 22613501

[B6] Gardin JM Leifer ES Fleg JL Whellan D Kokkinos P Leblanc MH Wolfel E Kitzman DW HF-ACTION Investigators. Relationship of Doppler-Echocardiographic left ventricular diastolic function to exercise performance in systolic heart failure: the HF-ACTION study Am Heart J 2009 158 S45 52 10.1016/j.ahj.2009.07.015 19782788 PMC2950162

[B7] Chattopadhyay S Alamgir MF Nikitin NP Rigby AS Clark AL Cleland JG Lack of diastolic reserve in patients with heart failure and normal ejection fraction Circ Heart Fail 2010 3 35 43 10.1161/CIRCHEARTFAILURE.108.824888 19850696

[B8] Leong DP Grover S Molaee P Chakrabarty A Shirazi M Cheng YH Penhall A Perry R Greville H Joseph MX Selvanayagam JB Nonvolumetric echocardiographic indices of right ventricular systolic function: validation with cardiovascular magnetic resonance and relationship with functional capacity Echocardiography 2012 29 455 63 10.1111/j.1540-8175.2011.01594.x 22176387

[B9] Rubis P Podolec P Tomkiewicz-Pajak L Kopec G Olszowska M Tracz W Usefulness of the evaluation of isovolumic and ejection phase myocardial signals during stress echocardiography in predicting exercise capacity in heart failure patients Echocardiography 2009 26 1050 9 10.1111/j.1540-8175.2009.00922.x 19840070

[B10] Conraads VM Metra M Kamp O De Keulenaer GW Pieske B Zamorano J Vardas PE Böhm M Dei Cas L Effects of the long-term administration of nebivolol on the clinical symptoms, exercise capacity, and left ventricular function of patients with diastolic dysfunction: results of the ELANDD study Eur J Heart Fail 2011 [Epub ahead of print] 10.1093/eurjhf/hfr161 22147202

[B11] Packer M Coats AJ Fowler MB Katus HA Krum H Mohacsi P Rouleau JL Tendera M Castaigne A Roecker EB Schultz MK DeMets DL Effect of carvedilol on survival in severe chronic heart failure N Engl J Med 2001 344 1651 8 10.1056/NEJM200105313442201 11386263

[B12] Grundtvig M Gullestad L Hole T Flønæs B Westheim A Characteristics, implementation of evidence-based management and outcome in patients with chronic heart failure: results from the Norwegian heart failure registry Eur J Cardiovasc Nurs 2011 10 44 9 10.1016/j.ejcnurse.2010.04.001 20452825

[B13] Bax JJ Bleeker GB Marwick TH Molhoek SG Boersma E Steendijk P van der Wall EE Schalij MJ Left ventricular dyssynchrony predicts response and prognosis after cardiac resynchronization therapy J Am Coll Cardiol 2004 44 1834 1840 10.1016/j.jacc.2004.08.016 15519016

[B14] Bajraktari G Dini FL Fontanive P Elezi S Berisha V Napoli AM Ciuti M Henein M Independent and incremental prognostic value of Doppler-derived left ventricular total isovolumic time in patients with systolic heart failure Int J Cardiol 2011 148 271 5 10.1016/j.ijcard.2009.09.567 19948365

[B15] Cleland JG Coletta AP Yassin A Buga L Torabi A Clark AL Clinical trials update from the European Society of Cardiology Meeting 2009: AAA, RELY, PROTECT, ACTIVE-I, European CRT survey, German pre-SCD II registry, and MADIT-CRT Eur J Heart Fail 2009 11 1214 9 10.1093/eurjhf/hfp162 19926603

[B16] Hunt SA Abraham WT Chin MH ACC/AHA 2005 Guideline Update for the Diagnosis and Management of Chronic Heart Failure in the Adult: a report of the American College of Cardiology/American Heart Association Task Force on Practice Guidelines (Writing Committee to Update the 2001 Guidelines for the Evaluation and Management of Heart Failure): developed in collaboration with the American College of Chest Physicians and the International Society for Heart and Lung Transplantation: endorsed by the Heart Rhythm Society Circulation 2005 112 e154 235 10.1161/CIRCULATIONAHA.105.167586 16160202

[B17] Bruch C Grude M Muller J Breithardt G Wichter T Usefulness of tissue Doppler imaging for estimation of left ventricular filling pressures in patients with systolic and diastolic heart failure Am J Cardiol 2005 95 892 5 10.1016/j.amjcard.2004.12.017 15781027

[B18] Gottdiener JS Bednarz J Devereux R American Society of Echocardiography. American Society of Echocardiography recommendations for use of echocardiography in clinical trials J Am Soc Echocardiogr 2004 17 1086 119 15452478 10.1016/j.echo.2004.07.013

[B19] Crapo RO Casaburi R Coates AL Enright PL MacIntyre NR McKay RT Johnson D Wanger JS Zeballos RJ Bittner V Mottram C ATS Statement: guidelines for the six-minute walk test Am J Respir Crit Care Med 2002 166 1111 1117

[B20] Ingle L Rigby AS Carroll S Butterly R King RF Cooke CB Cleland JG Clark AL Prognostic value of the 6 min walk test and self-perceived symptom severity in older patients with chronic heart failure Eur Heart J 2007 28 560 8 17314108 10.1093/eurheartj/ehl527

[B21] Rostagno C Olivo G Comeglio M Boddi V Banchelli M Galanti G Gensini GF Prognostic value of 6-min walk corridor test in patients with mild to moderate heart failure: comparison with other methods of functional evaluation Eur J Heart Fail 2003 5 3 247 52 10.1016/S1388-9842(02)00244-1 12798821

[B22] Bajraktari G Elezi S Berisha V Lindqvist P Rexhepaj N Henein MY Left ventricular asynchrony and raised filling pressure predict limited exercise performance assessed by 6 min walk test Int J Cardiol 2011 146 385 9 10.1016/j.ijcard.2009.07.018 19699003

[B23] Höglund C Alam M Thorstrand C Atrioventricular valve plane displacement in healthy persons An echocardiographic study. Acta Med Scand 1988 224 557 62 10.1111/j.0954-6820.1988.tb19626.x 3207068

[B24] Appleton CP Hatle LK Popp RL Relation of transmitral flow velocity patterns to left ventricular diastolic function: new insights from a combined hemodynamic and Doppler echocardiographic study J Am Coll Cardiol 1988 12 426 40 10.1016/0735-1097(88)90416-0 3392336

[B25] Duncan AM Francis DP Henein MY Gibson DG Importance of left ventricular activation in determining myocardial performance (Tei) index: comparison with total isovolumic time Int J Cardiol 2004 95 211 7 10.1016/j.ijcard.2003.07.007 15193822

[B26] Tei C Ling LH Hodge DO Bailey KR Oh JK Rodeheffer RJ Tajik AJ Seward JB New index of combined systolic and diastolic myocardial performance: a simple and reproducible measure of cardiac function - a study in normals and dilated cardiomyopathy J Cardiol 1995 26 357 366 8558414

[B27] Zoghbi WA Enriquez-Sarano M Foster E Grayburn PA Kraft CD Levine RA Nihoyannopoulos P Otto CM Quinones MA Rakowski H Stewart WJ Waggoner A Weissman NJ American Society of Echocardiography. Recommendations for evaluation of the severity of native valvular regurgitation with two-dimensional and Doppler echocardiography J Am Soc Echocardiogr 2003 16 777 802 10.1016/S0894-7317(03)00335-3 12835667

[B28] Gardin JM Adams DB Douglas PS Feigenbaum H Forst DH Fraser AG Grayburn PA Katz AS Keller AM Kerber RE Khandheria BK Klein AL Lang RM Pierard LA Quinones MA Schnittger I American Society of Echocardiography. Recommendations for a standardized report for adult transthoracic echocardiography: a report from the American Society of Echocardiography’s Nomenclature and Standards Committee and Task Force for a Standardized Echocardiography Report J Am Soc Echocardiogr 2002 15 275 90 10.1067/mje.2002.121536 11875394

[B29] Guyatt GH Sullivan MJ Thompson PJ Fallen EL Pugsley SO Taylor DW Berman LB The 6-min walk test: a new measure of exercise capacity in patients with chronic heart failure Can Med Assoc J 1985 132 919 23 3978515 PMC1345899

[B30] Guyatt GH Thompson PJ Berman LB Sullivan MJ Townsend M Jones NL Pugsley SO How should we measyre function in patients with chronic heart and lung disease? J Chronic Dis 1985 28 517 24 4008592 10.1016/0021-9681(85)90035-9

[B31] Chung ES Leon AR Tavazzi L Sun JP Nihoyannopoulos P Merlino J Abraham WT Ghio S Leclercq C Bax JJ Yu CM Gorcsan J 3rd St John Sutton M De Sutter J Murillo J Results of the Predictors of Response to CRT (PROSPECT) trial Circulation 2008 117 2608 16 10.1161/CIRCULATIONAHA.107.743120 18458170

[B32] Ingle L Rigby AS Nabb S Jones PK Clark AL Cleland JG Clinical determinants of poor six-minute walk test performance in patients with left ventricular systolic dysfunction and no major structural heart disease Eur J Heart Fail 2006 8 3 321 5 10.1016/j.ejheart.2005.08.006 16266825

[B33] Ritzema JL Richards AM Crozier IG Frampton CF Melton IC Doughty RN Stewart JT Eigler N Whiting J Abraham WT Troughton RW Serial Doppler echocardiography and tissue Doppler imaging in the detection of elevated directly measured left atrial pressure in ambulant subjects with chronic heart failure JACC Cardiovasc Imaging 2011 4 927 34 10.1016/j.jcmg.2011.07.004 21920328

[B34] Gehlbach BK Geppert E The pulmonary manifestations of left heart failure Chest 2004 125 669 82 10.1378/chest.125.2.669 14769751

[B35] Lindqvist P Mörner S Henein MY Cardiac mechanisms underlying normal exercise tolerance: gender impact Eur J Appl Physiol 2012 112 451 9 10.1007/s00421-011-1992-2 21584684

[B36] Foley PW Patel K Irwin N Sanderson JE Frenneaux MP Smith RE Stegemann B Leyva F Cardiac resynchronisation therapy in patients with heart failure and a normal QRS duration: the RESPOND study Heart 2011 97 1041 7 10.1136/hrt.2010.208355 21339317

[B37] Borlaug BA Paulus WJ Heart failure with preserved ejection fraction: pathophysiology, diagnosis, and treatment Eur Heart J 2011 32 670 9 10.1093/eurheartj/ehq426 21138935 PMC3056204

[B38] Kuppahally SS Michaels AD Tandar A Gilbert EM Litwin SE Bader FM Can echocardiographic evaluation of cardiopulmonary hemodynamics decrease right heart catheterizations in end-stage heart failure patients awaiting transplantation? Am J Cardiol 2010 106 1657 62 10.1016/j.amjcard.2010.07.022 21094370

